# Research progress on treating urinary system stones using ureteroscopy combined With a negative-pressure suction sheath

**DOI:** 10.3389/fsurg.2026.1840426

**Published:** 2026-05-15

**Authors:** Junqiang Liang, Hui Xiao, Song Yu

**Affiliations:** Department of Urology, The First People’s Hospital of Chuzhou City, Chuzhou City, Anhui, China

**Keywords:** intrarenal pressure, irrigation dynamics, negative-pressure suction sheath, ureteral access sheath, ureteroscopy, urosepsis

## Abstract

Ureteroscopy, which is now one of the primary treatments for kidney and ureteral stones, can suffer from unstable vision due to debris and fluid retention, fragment and dust migration, and temporary increases in intrarenal pressure resulting in infectious complications in vulnerable patients. Negative-pressure suction sheaths were introduced as an aid to ureteroscopy to assist in evacuating debris and turbid irrigant while providing more consistent flow dynamics. These devices are intended to improve both the efficiency of the procedure and the physiological safety of the patients. This review will combine the current research on ureteroscopy using suction sheaths with the integration of device design, irrigation-suction coupling, technical standardization, and clinical outcome data. Stone characteristics, device specifications, irrigation/suction variables, and primary endpoint data including stone-free rates, operative time, residual fragment burden, postoperative pain, ureteral injuries, and infectious complications (fever, SIRS, sepsis) were extracted from all eligible studies. The evidence is presented within a mechanistic to outcome model, where suction-assisted flow improves optical clarity, facilitates fragment evacuation, and reduces pressure surges during high energy lithotripsy. The model also addresses tradeoffs related to mucosal collapse risks, ureteral wall stresses, and the requirement for parameter standardization. Suction-assisted ureteroscopy provides a systems-based approach to improving the safety and efficacy of endoscopic lithotripsy, potentially most beneficially applied to high-dusting cases, contaminated or obstructed collecting systems, and anatomically challenging situations. To facilitate the widespread acceptance of this technology, there should be standardized reporting of intrarenal pressure surrogates, suction variable ranges, and comparison techniques used in conjunction with large-scale multicenter clinical trials, and cost-effectiveness analyses.

## 1 Introduction

### 1.1 Global and regional burden of urinary system stones and procedural trends

The urinary system stones are an extremely prevalent and recurrent condition; the associated clinical and economic burden is substantial due to acute colic, obstruction, infection, chronic pain syndrome and multiple interventions. In addition, the prevalence is increasing globally for reasons that include metabolic risk factor clustering, dietary changes, climate-related dehydration and advances in imaging technology (1–4). The trend toward less invasive treatment modalities (endourology) for urinary tract stones is a result of their ability to provide rapid relief of symptoms, shorter lengths of stay in hospitals, and wide applicability to all locations within the urinary system (5–8). Ureteroscopy, especially flexible ureteroscopy with laser lithotripsy, has evolved from being primarily used as a procedure for the ureters to a primary method of management for intrarenal calculi, even when patients were previously considered for shock wave lithotripsy or percutaneous nephrolithotomy (9–13). With this shift to flexible ureteroscopy comes a new level of expectation: higher success rates for removal of the stone with fewer residual fragments, lower infectious complication rates, and a predictable duration of operative time with consistent results despite varying anatomy and stone composition. Despite the need for and the desire to meet these performance standards, numerous obstacles exist in the practice setting that will impede procedural efficiency, increase the risk of complications to the patient, and ultimately affect the likelihood of additional treatments required. These barriers to efficient care delivery include obscured visual fields during active fragmentation, the buildup of particulate matter (dust), and micro-fragments during the workflow, stone migration and retropulsion, and increased intrarenal pressure during irrigation that may lead to pyelovenous and pyelolymphatic backflow. Therefore, there exists a significant opportunity for additional technological innovations to support endoscopic irrigation-based practices; specifically to convert the current passive, pressure-driven irrigation environment to a controlled system where the factors affecting visual clarity (visibility), fluid flow (outflow), and evacuation of fragments are systematically designed and controlled, vs. being developed on an ad-hoc basis.

### 1.2 Evolution of ureteroscopy and ureteral access sheaths: from passive outflow to active evacuation

To understand the development of ureteroscopy as a sequence of convergence of various technologies (optics, deflection mechanisms, channel efficiency, and energy delivery) that enable progressive reduction in profile size and increased durability of the instrument for precise intraluminal interventions. As well, there was an advancement of access and outflow systems that have transitioned from repeated ureteral passages (cumulative damage to mucosa and time spent) to the use of ureteral access sheaths which allow for repeated scope exchanges (reduced friction), provide improved irrigation flow and lower intrarenal pressure than no-sheath conditions under similar irrigation conditions (14–18). However, the currently available conventional ureteral access sheaths are essentially passive conduit devices, whose performance is dependent upon the user's irrigation technique, sheath-scope interface clearance, and the hydrodynamic properties of the drainage path; they also do not ensure directed transport of particles and/or fragments, and ensure pressure control during periods of high flow irrigation and prolonged lithotripsy (19–21).

Negative-pressure suction sheaths represent a paradigmatic shift from passive outflow facilitation to active removal of fluid and solids (22–26). Suction is applied at the point-of-care to remove turbid irrigants, blood-tinged effluents, bacteria-laden urine, and particulate debris created during lithotripsy procedures. This paradigmatic shift has far-reaching implications beyond the realm of operational convenience; the application of suction sheaths enables active management of the equilibrium between inflow and outflow of fluid into/out of the renal collecting system, potentially reducing peak intrarenal pressures generated by the suction process, shortening the time for which the urothelial surface is exposed to potential contaminants within the irrigation fluid, and improving the optical quality of the viewing environment for endoscopic manipulation of the ureteral lumen.

### 1.3 Pathophysiology link: irrigation, intrarenal pressure, backflow, and infection risk

The collecting system during ureteroscopy is a continuously changing hydraulic chamber (27–29). The choice of irrigation influences how much fluid flows in and out of the collecting system. The interaction between anatomic resistance, the presence of obstruction, the renal pelvis and calyceal compliance, and available pathways for clearance via the ureter and/or an access sheath will all influence pressure-flow dynamics (30–33). When the flow in is more than the flow out, the pressure within the renal collecting system increases. Transient increases in pressure may occur without adverse consequences in many patients. However, when bacteriuria is present and there is elevated pressure in the setting of infected stones or pyonephrosis, backflow mechanisms are facilitated to allow endotoxins to enter the systemic circulation, thus linking intraoperative hydraulic conditions to postoperative fever, SIRS and sepsis (34–38). Increased pressure and turbulent effluent in non-infected cases can also contribute to worsening bleeding and impaired visualization during ureteroscopy, resulting in prolonged operative times and increased heat load during laser lithotripsy due to inefficient thermal distribution. Repeated interruptions for clearing the visual field also increase the overall duration of the procedure. Residual fragments and dust are not inert products of fragmentation and are associated with recurrent risk and additional downstream interventions. Poor evacuation during the process of removing dust and debris can result in a clinically relevant “stone persistence” even if the initial fragmentation was technically successful (39–42). Therefore, suction-assisted methods attempt to modulate the linked mechanisms as a single entity by improving the transport of debris from the kidney while maintaining a stable pressure profile. Therefore, the assessment should include not only the stone-free rate, but also the intermediate mechanisms of success (e.g., visibility stability, irrigation volume, pressure surrogate, operative time, infection related outcomes) that collectively impact the patient outcomes. In keeping with this paradigm, the present review integrates fluidic logic with clinical outcomes, as opposed to simply viewing suction as an additive treatment, since the ultimate clinical benefit of negative-pressure suction sheaths will depend upon their ability to alter the endoscopic micro-environment in meaningful ways that promote safer, more complete, and more efficient removal of stones.

### 1.4 Rationale for negative-pressure suction sheaths as a systems-level adjunct

The use of negative-pressure suction sheaths represents an example of a system-based approach to modifying three operational bottlenecks simultaneously: optical clarity, removal of fragments, and pressure dynamics within the renal pelvis. By continually aspirating turbid effluent and particulate matter from the field of view, suction can provide for stable conditions for viewing during procedures, while reducing the amount of time spent clearing the visual axis using repeated flushing, basketing or scope withdrawal. In addition, suction sheaths facilitate directional transport of particulate and fragment debris through controlled outflow thereby potentially decreasing the number of debris present post-operatively. This could result in improved stone-free rates when used in conjunction with a workflow that relies heavily upon dusting to remove small fragments and/or micro-fragments. Suction sheaths may also aid in minimizing the risk of infection associated with excessive pressure fluctuations in the inflow-outflow pathway, especially in high-risk scenarios including infected stones, obstruction of the urinary collecting system(s) and/or extended operative times. Nonetheless, the ability of suction sheaths to realize these potential benefits are dependent upon several factors, including design characteristics of the suction sheath (e.g., diameter of the sheath, internal geometry of the lumen, number and size of fenestrations, valve behavior); suction modality (i.e., continuous or intermittent, range of vacuum, rate of flow); method of irrigation (i.e., gravity-flow vs. pressurized-flow; rate of flow); and procedural characteristics (size, location, anatomy, etc. of the stone being treated; technical proficiency of the surgeon/operator). Due to the heterogeneity of suction devices currently available and variability in how the literature reports data, a detailed systematic analysis is required to determine which suction device design configurations have been demonstrated to be beneficial, what trade-offs occur among various design options and where uncertainty exists regarding the efficacy of specific designs. Therefore, this review is organized along a mechanistic-to-clinical continuum to enable readers to understand the relationship between the outcome of interest (e.g., success rate, complication rate) and the underlying physiological/fluidic processes are involved. Additionally, this review will provide insight into which design variable(s) should be standardized across studies to enable comparisons and facilitate clinical translation.

### 1.5 Clinical significance and patient-centered decision impact

The clinical importance of suction assisted ureteroscopy lies in its ability to alter the operating performance of ureteroscopy when the conventional technique of ureteroscopy has reached a plateau: Large numbers of renal stones being removed using prolonged “dusting”, poor visualization due to bleeding or heavy particulate load, and complicated anatomy (i.e., the “time” costs associated with these cases) and those with a high risk of infection where the containment of pressure and removal of contaminated fluid will be especially important. For suction to provide a reliable reduction in operative time with equal or improved stone free rates, it could also provide a reduction in anesthetic time and resource use, and if it can provide a reduction in postoperative infections in high risk populations, which will help solve one of the major complications of stone surgery. However, if suction provides additional risks such as increased stress on the ureteral wall from increased diameter sheaths, mucosa apposition from excessive negative pressure, or operational complexity that causes variability in workflow, then suction should be adopted based on standardization of parameters and evidence rather than just enthusiasm. For that reason, the purpose of this literature review is to guide both clinicians and researchers. The purpose of clinicians is to identify which patient populations and operative environments are most likely to benefit from suction assisted ureteroscopy. The purpose of researchers is to provide a list of necessary elements for comparative reporting including suction settings, irrigation mode, sheath – scope clearance, surrogate or direct measures of intrarenal pressures, microbial end points, and standardized definitions for stone free status and residual fragments. By providing a mechanistic rationale supported by a structured synthesis of existing literature and identifying areas of need for further study, this literature review aims to support the continued development of suction assisted ureteroscopy as a mature and reproducible procedure that can be evaluated with the same level of scrutiny as other high impact clinical innovations.

## 2 Review methodology and literature search

A structured literature review was conducted across PubMed, Embase, Web of Science, Cochrane Library, CNKI, Wanfang, and ClinicalTrials.gov. Searches targeted studies published from January 2020 to October 2025 and combined terms related to ureteroscopy procedure, negative pressure/suction sheath technique, outcome/safety of stone disease by using Boolean operators AND/OR. English-language and Chinese-language publications were considered.

Eligible records included original preclinical studies, clinical studies, translational studies, and high-quality reviews that were directly relevant to ureteroscopy procedure, negative pressure/suction sheath technique. Editorials, conference abstracts without usable data, duplicate reports, and studies without sufficient methodological detail were excluded. Titles and abstracts were screened first, followed by full-text assessment of potentially eligible studies. The specific eligibility criteria are shown in Table 1.

**Table 1:** Eligibility criteria.

Inclusion Criteria	Exclusion Criteria
Human studies evaluating ureteroscopy (rigid, semirigid, flexible) for ureteral and/or renal stones with an active negative-pressure suction/aspiration sheath or sheath-like suction system used intraoperatively	Non-human (bench-only, animal-only) studies without a clinically interpretable linkage to ureteroscopic suction-sheath use
Randomized trials, nonrandomized comparative studies, prospective/retrospective cohorts, and sufficiently detailed case series when comparative evidence is limited; conference abstracts included only if outcomes and device parameters are extractable	Case reports with insufficient procedural detail, narrative opinions, letters without primary data, and abstracts lacking extractable outcomes or device/suction parameters
Studies reporting at least one clinically relevant endpoint (stone-free status, residual fragments, operative time, complications including infection/fever/sepsis, ureteral injury, readmission, pain) or mechanistic endpoints (intrarenal pressure measures/surrogates, visibility proxies, irrigation/suction parameters)	Studies where suction is used outsideureteroscopic stone surgery (e.g., percutaneous-only platforms) or where the intervention is not a ureteroscopic suction sheath (e.g., simple basket aspiration without suction device context)
Adult populations and mixed-age cohorts; pediatric/adolescent cohorts included if ureteroscopic suction-sheath use and outcomes are clearly delineated	Purely pediatric-only reports if anatomy/device constraints render findings non-transferable and outcomes cannot be contextualized within the broader evidence base
English-language full texts and non-English studies with accessible full translations/structured data allowing accurate extraction	Non-English studies without a reliable translation or incomplete reporting that prevents valid extraction and risk-of-bias appraisal
Studies in any clinical setting provide perioperative pathways and techniques that are described with sufficient clarity (stone metrics, device type, suction/irrigation approach, comparator where relevant)	Duplicate cohorts where outcomes are fully overlapping; in such cases, the most comprehensive dataset is retained and companion reports used only for non-duplicative details

**Figure 1:**
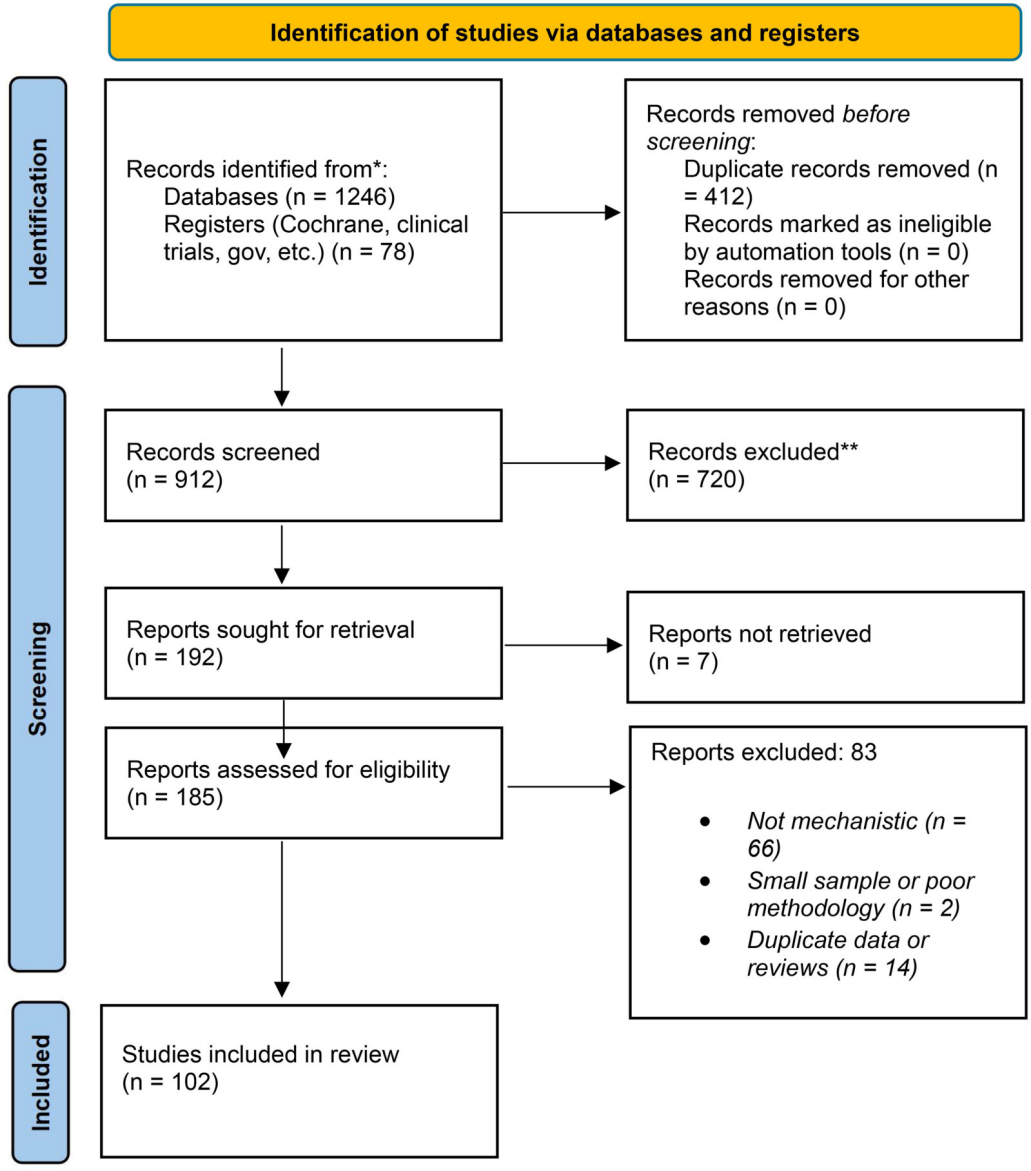
PRISMA flow diagram for study identification and selection.

Because the available evidence was heterogeneous and frequently incomplete with respect to dose, comparator design, outcome definition, and effect-size reporting, the revised manuscript uses a critical narrative synthesis rather than a formal meta-analysis. Risk-of-bias tools such as RoB 2 and ROBINS-I are appropriate for future study-level appraisal when a finalized evidence set is locked for submission. The PRISMA diagram can be viewed as Figure 1 below.

### 2.1 Data extraction and quality appraisal

The following is a description of how the authors obtained study level and intervention level variables that align with the “mechanism to clinic” logic of SU by applying a systematic framework for extracting study data. Variables of interest include demographic information about the patient population including markers of risk (such as infection markers when available), descriptive characteristics of the stones being treated (such as size, number, location and proxies for density/composition) and procedural aspects (such as rigid, semirigid flexible ureteroscopy, RIRS designation, laser platforms and strategies when identified) and device characteristics (such as sheath diameter, device type and architecture, suction on/off intensity if reported, irrigation modalities and strategies and whether there was monitoring of intrarenal pressures or surrogates). The authors also extracted outcomes into pre-determined categories such as effectiveness (definition of “stone free”, timing of stone free status, threshold for residual fragments, rate of retreatment), efficiency (operative time, amount of fluoroscopy, length of hospital stay) and safety (presence of fever, SIRS or sepsis, presence of signs of ureteral injury/ stricture, hematuria, post-operative pain, need for readmission). The authors also assessed the risk of bias in each study using appropriate tools based upon the design of each study (domains specific to randomized trials for RCTs and selection/confounding/measurement domains for observational studies); they placed additional emphasis on identifying potential confounders related to technique (e.g., laser strategy, basket use, stenting practices), variable definitions regarding outcomes (specifically imaging modality and time point for stone free determination) and potential selective reporting of suction and/or irrigation parameters due to their direct impact on interpreting results from studies with varying methodology and their importance in establishing evidence at high levels of certainty.

### 2.2 Synthesis of study characteristics and heterogeneity

Although a formal comparative table is not presented, the key characteristics and methodological landscape of the studies informing this review are synthesized descriptively below to provide context for the mechanistic and outcome discussions that follow. The evidence base primarily comprises recent (2020–2025) comparative cohort studies and a limited number of randomized controlled trials (RCTs), reflecting the emerging clinical adoption of suction-assisted ureteroscopy (SAU). A recurring methodological challenge across these studies is the heterogeneity in design and reporting, which directly motivates the standardization framework proposed later in this review. The study populations largely focused on adults with renal or proximal ureteral stones, with stone burdens often categorized as moderate to high, or in scenarios predicting significant dust generation. A notable subset of studies explicitly enrolled patients deemed at higher risk for infectious complications, such as those with preoperative hydronephrosis, positive urine cultures, or suspected infected stones, aiming to test the pressure-control rationale for SAU. The most significant source of heterogeneity lies in the intervention itself. Studies evaluated different commercial iterations of suction sheaths, varying in caliber, internal design, and the mechanism of suction application (continuous, intermittent, or surgeon-modulated). This was coupled with variable irrigation protocols and laser lithotripsy settings (dusting vs. fragmenting). Crucially, parameters such as the exact negative pressure applied (mmHg) or flow rates (cc/min) were frequently under-reported, making precise technical replication difficult. Regarding outcomes, definitions for “stone-free” status were inconsistent, employing different imaging modalities at varying postoperative timepoints with non-uniform fragment size thresholds. Reporting of infectious complications was also not standardized, with variable adherence to consensus definitions and differing protocols for perioperative antibiotic use and culture acquisition. Safety reporting for ureteral injuries often lacked graded severity scales.

This descriptive synthesis underscores that while the collective evidence points toward the mechanistic benefits and clinical utility of SAU—particularly in improving visibility, reducing intrarenal pressure surges, and potentially lowering infection risk—the variability in “who was treated, with what exact technique, and how outcomes were measured” fundamentally limits quantitative meta-analysis. The following sections therefore interpret findings through this lens of methodological diversity, and the subsequent call for standardization aims directly at resolving these inconsistencies to enable more definitive comparative synthesis in the future.

## 3 Device concepts

A ureteroscopy uses ureteroscopic negative-pressure suction sheaths to create a controlled flow circuit for inflow, outflow and particulate transport (43). The primary purpose of conventional ureteral access sheaths have been used to provide a low resistance path for repeated scope exchanges and improve passive egress of irrigating solution (14). They do not inherently control the direction of dust transport or consistently prevent intrarenal pressure increases with increasing irrigant flows to maintain visibility during high energy lithotripsy. By creating a controlled active negative pressure drains at the distal end of the sheath, these suction enabled devices intend to maintain a clear visual field, increase the rate of removal of suspended debris and blood tinged effluent and stabilize renal pelvic pressures by maintaining a consistent net outflow as compared to inflow (10, 44, 45). The desired effects will occur through several recurring design variables common to all device families: diameter and length of the sheath (lumen resistance and placement stability), internal lumen design (single lumen with scope clearance vs. coaxial or partitioned pathway) distal tip geometry and fenestrations (capture particulates and apposition potential), and the mechanical components used to seal and/or valve the sheath to maintain a negative pressure environment while providing mobility to the scope. Importantly, “negative pressure” is only clinically relevant if it creates a predictable net flow state. Aspiration of suction may be delivered continuously to provide a steady washout, intermittently to minimize the risk of mucosal apposition when performing delicate calyceal work, or adaptively based upon the level of turbidity and dust load (46–48). The choice of modalities in this regard is not simply a matter of technical preference, but rather determines the local hydraulic conditions governing the visible area, heat dissipation and fragment residence time, and therefore should be considered as part of the procedure, rather than ancillary.

A good mechanical model is to consider the pressure gradient created by the suction-assisted ureteroscopy as being controlled. This creates a bias towards the movement of fluids and particles away from the kidney through the collecting system while minimizing the amount of pressure that is developed. The renal pressure generated can be thought of as the difference between the inflow pressure and the outflow capacity (49, 50). The outflow capacity is limited by the resistance of the ureter, the size of the instrument occupying the ureter, the clearance between the instrument sheath and the ureteroscope, the bends in the ureter and the intermittent blockage caused by fragments of stones (51). Conventional flexible ureteroscopes, when used with the technique of dusting (dusting refers to the process of using the ureteroscope to break up stones into smaller pieces), suspended particles cause turbidity and are likely to create stagnant pools of fluid in lower portions of the collecting system which will encourage recirculation and therefore extend the duration of the procedure and the need to increase irrigation pressure to maintain visualization (40, 52, 53). Suction removes suspended particles and can improve the advective removal of these particles and reduce both the average and maximum renal pressures for the same irrigating strategy thus improving the two interrelated problems of optical clarity and pressure variability. The physical principles used to explain the effects of suction on the physics of flexible ureteroscopy, also describe the potential failure modes. If the suction is greater than the inflow then local areas of negative pressure can pull urothelium onto the scope tip or sheath edge and produce intermittent “suction lock,” mucosal petechiae, bleeding, and a paradoxical decrease in visibility (54). If the inflow rate is decreased too much to maintain a low pressure environment, there may be a higher thermal risk associated with laser use, because of the lack of sufficient fluid exchange and poor heat dissipation. The ideal clinical range of operation for suction assisted ureteroscopy, is to provide a buffering zone, such that the irrigation maintains an adequate working space and thermal control, and the suction provides accelerated wash-out of the dust and waste products, thus reducing the degree of turbidity and dampening the pressure spikes, but not creating tissue apposition (55, 56).

Physiological safety considerations extend beyond generic lower-pressure assumptions and must include integration of infection biology with ureteral wall mechanics and thermodynamics micro-environment effects. Clinically relevant surges in pressure during ureteroscopy provide a plausible mechanisms for pyelovenous and pyelolymphatic backflow, especially in contaminated systems where bacteria, endotoxin, and inflammatory mediators may be present within the renal pelvic urine or colonized stones (33, 50, 57). The use of suction-assisted outflow has been hypothesized to reduce the probability and magnitude of such surges by maintaining outflow capacity ahead of episodic increases in inflow. This logic is most salient in obstructed or hydronephrotic systems where irrigation is repeatedly increased to restore visibility during prolonged lithotripsy. Thermal safety is closely coupled to flow; high-power laser activation in a confined aqueous space can generate clinically relevant heat and the effective mitigation is adequate fluid exchange near the laser-stone interface (58–61). Suction can be protective if it increases net turnover and prevents stagnation, but it can be harmful if it promotes low-flow conditions or encourages prolonged activation under visually “clear” but hydraulically low-turnover states. Tissue safety finally depends upon both insertion mechanics and intraluminal forces; larger sheaths can increase ureteral wall stress during placement in tight ureters, while overly aggressive suction can transiently increase mucosal shear and contact forces at the distal tip of the suction device. These safety dimensions indicate that suction-assisted ureteroscopy should be judged not as a binary device choice, but rather as an operating envelope defined by sheath design, placement feasibility, suction modality, irrigation strategy, and laser settings.

Because terminology and device implementations are inconsistent across the literature and across manufacturers, standardizing conceptual descriptors is essential for high-precision interpretation and for designing future trials that can meaningfully compare interventions. The following table captures the core mechanistic variables that determine performance and safety in suction-assisted ureteroscopy and provides a common language for subsequent sections focused on technique and clinical outcomes. As shown in Table 2 for details.

**Table 2:** The core mechanistic variables in suction assisted ureteroscopy.

Mechanistic domain	Core variable	Operational meaning in surgery	Expected benefit when optimized	Key tradeoff if poorly controlled
Geometry (62)	Sheath caliber and length	Governs luminal resistance, placement stability, scope mobility	Stable access with adequate outflow capacity	Larger caliber may increase placement trauma in tight ureters
Architecture (63)	Lumen design and sealing	Determines ability to sustain negative pressure while permitting scope motion	Predictable evacuation without loss of maneuverability	Poor seal reduces effective suction; excessive seal can hinder flow dynamics
Suction delivery (64)	Continuous vs intermittent vs adaptive	Controls effluent clearance dynamics and apposition risk	Stable visibility and washout tailored to dust load	Excess suction can cause mucosal apposition/bleeding and field instability
Irrigation coupling (65)	Gravity vs pump; inflow intensity	Sets baseline inflow pressure and thermal buffering	Adequate working space and heat dissipation with minimized pressure surges	Over-irrigation drives pressure spikes; under-irrigation increases thermal risk
Fragment transport (66)	Dust suspension and evacuation	Determines residence time of particulate and probability of residuals	Lower fine residual burden and faster completion	Stagnation promotes residual fragments; turbulence may worsen migration

**Figure 2:**
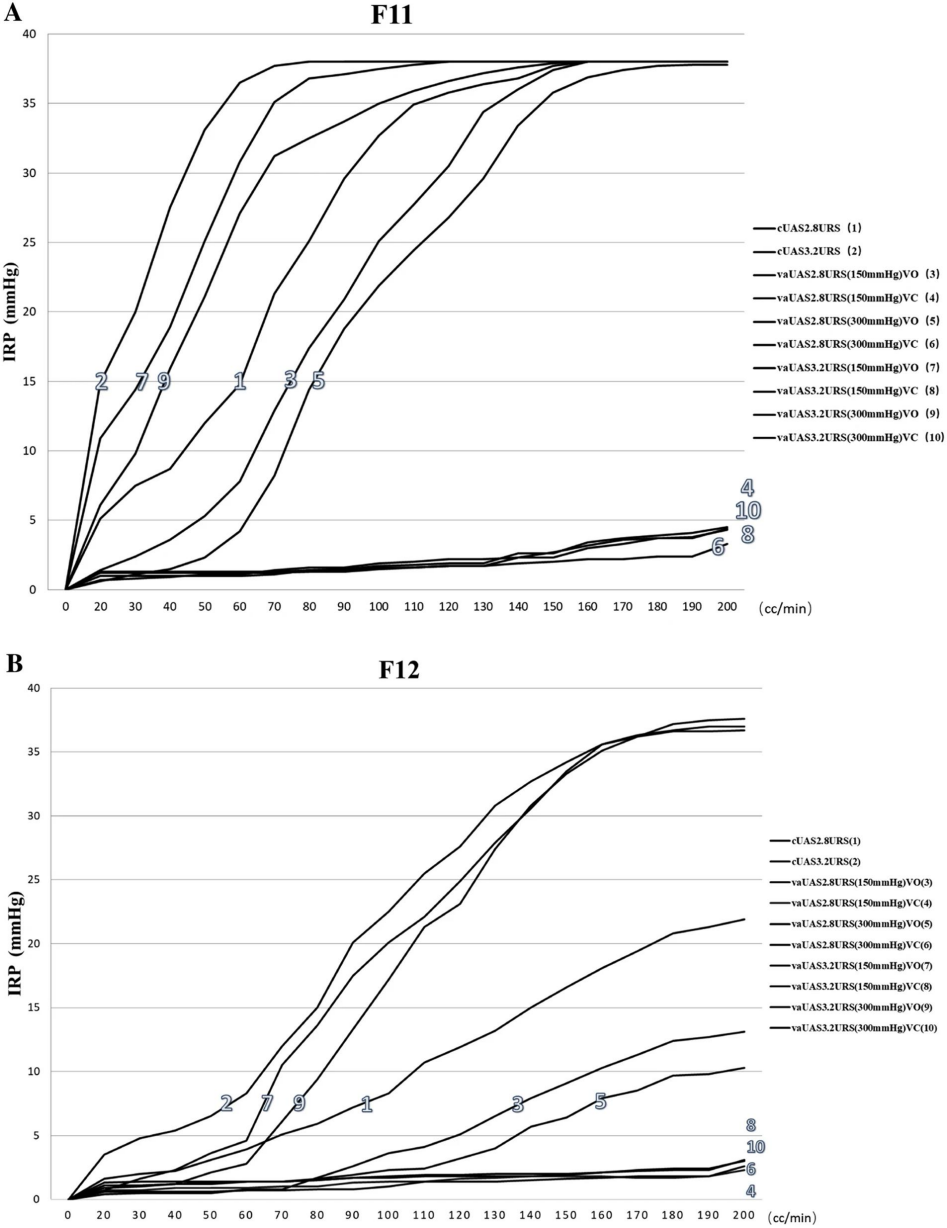
**A** the intergroup analysis of variation between intrarenal pressure and irrigation rates in 11Fr. ureteral access sheath. **B** The intergroup analysis of variation between intrarenal pressure and irrigation rates in 12Fr. ureteral access sheath (67).

Figure 2 shows the graphical representation of the relationship between intrarenal presssure and ureteral access sheath suction in flexible ureteroscopy (67).

## 4 Operative technique and perioperative management

Based on the fluid mechanics mechanisms and safety considerations mentioned earlier, this section aims to synthesize existing evidence and derive an optimized perioperative practice framework. The following description of patient selection, intraoperative procedures, and complication prevention integrates the principles supported by literature and the clinical recommendations proposed by the author's team based on mechanism understanding, aiming to provide a systematic reference for the safe and effective implementation of suction assisted ureteroscopy surgery. Successful use of suction during ureteroscopy with a suction sheath will depend on how well you can manage the three components of the “Fluid-Energy-Tissue” triangle and the relationship they have to each other. The fluid component refers to the flow of irrigation, which must provide adequate support to both stable visualization and heat dissipation (the ability to remove heat generated from the laser). Energy delivery must be managed in relation to the systems' clearance capacity. Tissue management must also be carefully managed due to the limitations in accessing the ureters. Therefore, it would be reasonable to develop a plan for preoperative evaluation of your patient using two intersecting axes, namely clearance complexity and physiological susceptibility, so that suction may be applied in areas that are most likely to have a high degree of success in terms of providing active removal of debris as part of the procedure. The clearance complexity of a stone is generally determined by the size of the stone burden, specifically those larger than the maximum diameter possible for passage through the ureter, and the number of stones present (multiples) and their location within the renal collecting system (calyx) (8, 68, 69). Stones located in the lower pole of the kidney have anatomical characteristics that favor the sedimentation of particles in the dependent aspect of the stone bed and create a mechanical disadvantage for maneuverability of instruments in the lower pole and the potential for sustained deflection of the ureteroscope. The expected fragility or type of stone, which is assumed based on the expected quantity of stone fragments produced by lithotripsy, determines if the procedure is going to be limited by the amount of particulate matter being produced rather than the actual rate at which stone fragments are created (70, 71). Susceptibility is determined by the patient's history of obstruction, hydronephrosis, previous instrumentation, current urine culture results, current levels of systemic inflammatory markers, and past medical history that indicates colonization of the stones, because all of these factors increase the severity of the consequences of increased intrarenal pressure and prolonged exposure to contaminated effluent. A third filter that must be evaluated concurrently is the feasibility of access to the ureter(s), since non-traumatic placement of the sheath can convert an optimization process into unnecessary ureteral injury; the patient's ureteral caliber, suspected stricture, prior ureteral surgery, and stent history should guide a low threshold for a decision to place a stent before proceeding with the ureteroscopy or stage the ureteroscopy if significant resistance is met during advancement of the ureteroscope.

The intraoperative workflow for suction assisted nephrolithotomy is based on sequential control of access stability and hydraulic stability to ensure safe high-dust lithotripsy (72, 73). When placing the sheath, it should be done using an atraumatic method of placement: advancing the sheath progressively with tactile sensation and avoiding applying force to tight areas of the ureter, and being prepared to remove the sheath if there is resistance that indicates a high risk of ureteral injury. After positioning the sheath, the surgeon needs to confirm functional stability by confirming that irrigation will produce rapid flow, and that suction will create controlled clearance, without intermittent collapse of the working space. Early instability of the working space typically will predict future complications including mucosal apposition, bleeding, and decreased visibility. Evidence suggests that relying on increasing perfusion pressure to improve visual clarity can lead to an increase in renal pressure. Based on this mechanism, we suggest an important shift in intraoperative practice: making adjusting attraction parameters the primary means of managing visual field clarity, rather than prioritizing increasing perfusion pressure. In traditional ureteroscopy, increased turbidity has been used to justify increasing the pump setting, which increases intrarenal pressure and thus risks for potential physiologic complications; with suction assisted nephrolithotomy, increased turbidity should be used to adjust the amount of suction titrated and strategically mobilize dust while maintaining low irrigation pressure to provide a buffer zone for clearing dust safely and thermally. Suction assisted nephrolithotomy approaches are especially useful during long periods of intrarenal dusting, where clearance of dust—not fragmentation capability—often limits the speed of the procedure, and where repeated escalations of pressure may compound the risk of infection in susceptible patients (74, 75). The laser strategy must take into account the downstream clearance pathway. The particulate generated by dusting is easily controlled when suction and irrigation are both utilized to create a suspension and directional transport of particles; however, when the flow conditions exist such that the particulate settles into dependent calyces and creates an appearance of endoscopic completeness, it results in residual fragments postoperatively (39). The fragmentation and extraction strategies will likely receive less direct benefit from suction regarding the removal of macroscopic particles; however, suction can help to stabilize the view, decrease the amount of time needed to clean the area, and help with the egress of small particles, especially if the view is intermittently occluded by bleeding or mucosal oozing. Suction should always be viewed as a variable factor as opposed to being a constant (i.e., suction can be continuously used at low to moderate levels to maintain a steady washout during high-dust phases, suction can be intermittently used to minimize the risk of mucosa apposition during close work in the calyceal system, and suction can be used in short bursts after intense laser use to remove particulate that has accumulated without maintaining long-term negative pressure on the distal tip). It is critical that the suction level does not encourage under-irrigation; the safest operating parameters include sufficient inflow to dissipate heat and create a consistent working environment, and utilize suction to prevent turbidity build-up and minimize the pressure spikes that could lead to pump escalation (76, 77).

Fragment management should be implemented as a well thought out plan for a logical sequence of events rather than as an unplanned afterthought. The utilization of “clearance cycles” during renal dusting, which is a combination of short-term suction improvement and controlled irrigation pulses, in addition to scope repositioning, allows for particulate debris to be dislodged from the dependent recesses of the kidney, reduces the likelihood of stagnation within the calyces, and will generally reduce the likelihood that residual fragments will remain at the conclusion of endoscopy even though it appears complete (78). In those instances where there is larger particulate material created, suction may help direct some of the smaller pieces towards egress; however, basketing is still necessary for removing clinically significant material that causes post-operative pain (colic), obstruction, or additional surgical interventions; in such cases, suction is primarily used to provide clarity and to stabilize pressure fluctuation during the time extraction is occurring (79, 80). Decisions regarding postoperative stenting should include consideration of ureteral handling, the relationship of sheath size to ureteral tolerance, operative duration, observed mucosal injury, and expected residual particulate burden (81–84). Although suction assistance may help shorten procedures, both sheath-based access and extended intrarenal manipulations can still result in temporary obstruction and edema, especially in higher burdened patients, and support a low threshold for stenting if safety signals arise. Infection prevention has to place suction-assisted ureteroscopy into the context of a perioperative pathway (as opposed to relying on suction as either a replacement for antimicrobial vigor or a first step before making a decision to drain an infected obstructed patient). Urine culture prior to surgery and treatment for specific bacteria are still key, but bladder cultures can often disagree with cultures obtained from the renal pelvis or from infected stones, and colonized stones can serve as a “reservoir” for pathogens even though the urine appears to be sterile at the time of the preoperative collection (85). A higher risk scenario includes recent infection, hydroureteronephrosis, multiple previous interventions, and suspicion of infected stones. Proximal urine samples in these patients will have meaningful microbiologic data, and they should be performed concurrently with a low-flow, intraoperative strategy that focuses on maintaining contained pressures. The most compelling argument for the use of suction in this manner would relate to its mechanical ability to reduce the need for high-flow irrigations, and thus limit the number of intrarenal pressure surges that could result in systemic spread of pathogens. However, if purulent effluent is noted during the procedure, or the patient begins to demonstrate physiologic instability, the primary objective of the procedure must then transition away from clearance of the obstructing stones and toward drainage and stabilization of the infected urinary tract. Continued lithotripsy in an infected urinary tract system is generally considered unsafe, regardless of the suction capabilities available (86).

**Figure 3:**
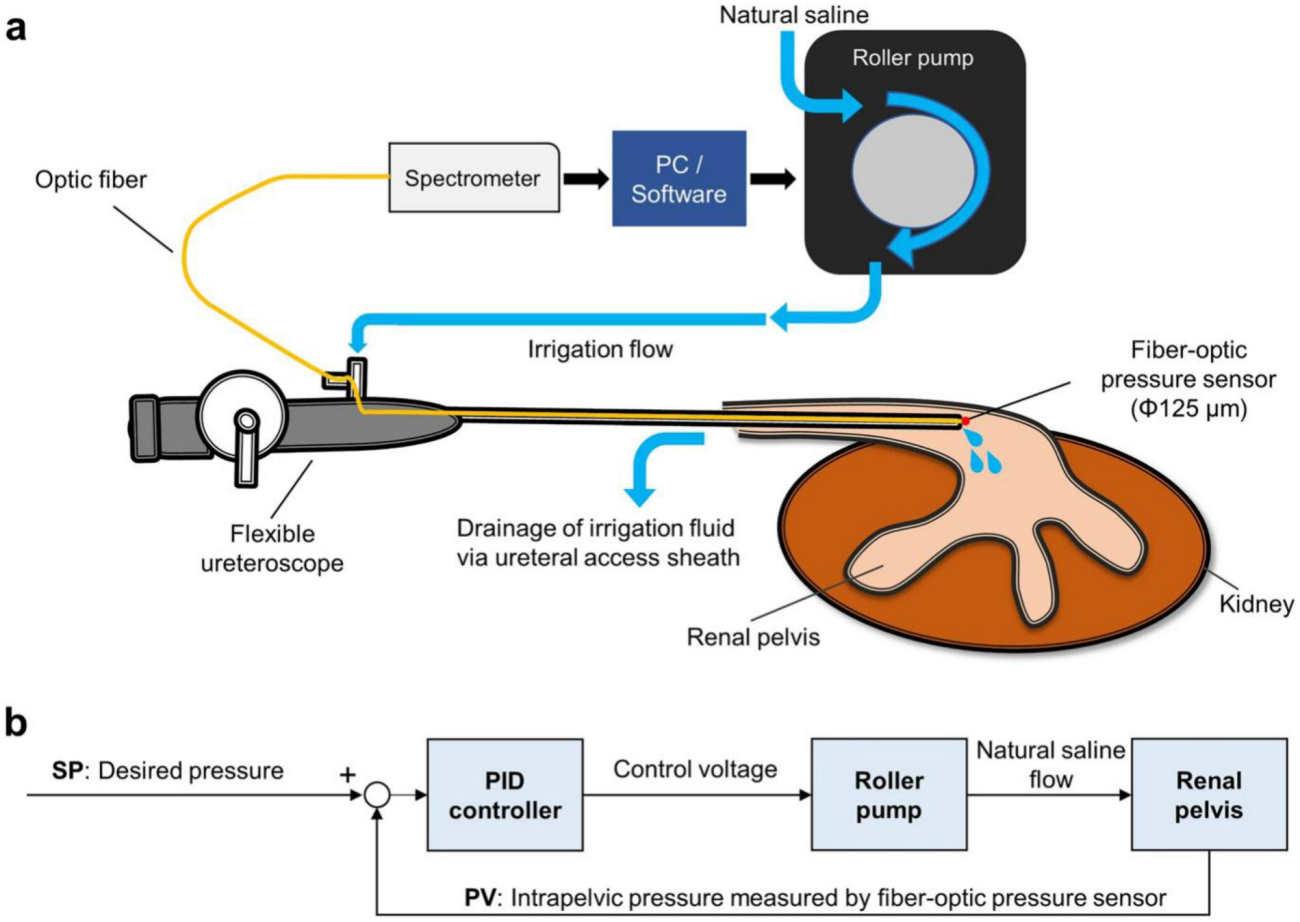
The concept of automatic irrigation system. **(a)** Scheme of the entire system. **(b)** The PID control block scheme. *PC* personal computer, *PID* proportional integral derivative, *PV* process variable, *SP* set point (87).

Figure 3 illustrates an example of an automatic irrigation system (87).

Suction-assisted ureteroscopy troubleshooting should be focused on achieving a stable flow as opposed to increasing force or pressure. The most common cause of failure to achieve adequate efflux using suction is related to some form of obstruction in the lumen (debris, kinked or poor seal dynamic) (88). Thus, when correcting for this issue, the operator will first verify tubing patency, then temporarily lower suction while flushing with controlled irrigation, pull back the endoscope slightly to relieve distal apposition, and finally assess the position of the sheath. If the operator encounters a “suction lock” due to intermittent mucosa, the operator should reduce suction, and transition to intermittent aspiration, and provide additional irrigation buffer to protect the working space; if the operator experiences persistent apposition, they should determine whether it would be safe to continue using suction at that particular anatomic site. As such, when the operator continues to have difficulty maintaining visualization, the operator should resist their initial reflex to dramatically increase irrigation pressure and instead utilize evacuation cycles and various techniques that decrease the dust generated from fragmentation such as adjusting laser parameters or alternately fragmenting and clearing. Finally, if the operator determines that sheath placement was not atraumatic or that there was significant ureteral resistance, then proceeding sheathless, placing a stent prior to performing staged ureteroscopy, or choosing an alternate method of treatment is safer and more likely to result in optimal outcomes than continuing to advance the sheath and risking further ureteral injury since the benefit of suction does not warrant unnecessary ureteral trauma. Various factors can refer to Table 3.

**Table 3:** Decision framework for safe and effective suction-assisted ureteroscopy.

Decision node	Primary objective	Key variables	Recommended operational principle	High-risk signal	Best-practice response
Preop case selection (42)	Use suction where it most improves safety/efficiency	Stone burden (size/multiplicity), location (esp. lower pole), anticipated dust load; obstruction/hydronephrosis, prior instrumentation, urine culture/inflammatory state	Select suction preferentially when dust/visibility will be rate-limiting and pressure containment matters	Lower-pole dominance + high dust + infection/obstruction vulnerability	Plan dynamic suction + clearance cycles; consider staging if burden/time-risk is high
Access feasibility/sheath safety gate (89)	Avoid ureteral injury from sheath placement	Ureteral caliber, tight segments/stricture suspicion, prior ureteral surgery, stent history, resistance on advancement	“Atraumatic feasibility overrides device preference” (do not force)	Resistance/tight ureter, suspected stricture, trauma signals	Proceed sheathless orpre-stent and stage rather than forcing sheath
Hydraulic regime (flow–pressure control) (27)	Maintain visibility + thermal safety while minimizing intrarenal pressure spikes	Inflow adequacy for heat dissipation, outflow behavior, working-space stability (collapse/apposition), turbidity trend	Achieve clarity via evacuation (suction), not irrigation escalation	Reflex pump escalation; intermittent collapse/apposition; persistent turbidity	Titrate suctionand keep irrigation at the lowest level that preserves a buffered working space
Energy–clearance matching (laser strategy) (56)	Prevent dust generation from exceeding evacuation capacity	Dusting vs fragmentation intent, activation cadence/settings vs observed clearance, dependent calyx sedimentation	Choose laser settings based on the downstream clearance pathway	“Looks complete” but dust settles in dependent calyx; prolonged activation with poor turnover	Introduce clearance cycles, reposition to mobilize debris, reduce dust rate if needed
Fragment management (clearance cycles + selective extraction) (90)	Reduce residual fragments and maintain field clarity	Dust accumulation in dependent recesses, visibility stability, fragment size distribution, need for basketing	Treat evacuation as a planned sequence(clearance cycles) and basket clinically meaningful fragments	Persistent turbidity; accumulating microfragments; risk-sized remnants	Periodic clearance cycles; mobilize/aspirate fine particulate; basketsignificant fragments
Infection pathway integration (57)	Prevent septic escalation and avoid pressure-chasing when flow fails	Preop culture mismatch risk, colonized stones suspicion; tubing patency, lumen obstruction/kink, mucosal apposition	Suction helps reduce high-pressure irrigation need, but does not replace antimicrobial/drainage logic; troubleshoot by restoring flow (not pressure)	Purulent efflux/physiologic instability; no efflux; suction lock/apposition	Check tubing/patency, flush gently, down-titrate suction to intermittent bursts, withdraw slightly; abort lithotripsy and drain if infection instability appears

## 5 Standardization framework for suction-assisted ureteroscopy

To overcome the heterogeneity of definitions and reports in current research and promote the development of the field, this article proposes the following standardized framework suggestions. The content of this chapter is not a description of existing consensus, but aims to provide a replicable reporting standard and classification system for future clinical trials, technological comparisons, and real-world research.

### 5.1 Terminology and intervention taxonomy

The main obstacle to “synthesizable” research on suction-assisted ureteroscopy is “definitional drift,” in other words, different types of approaches to suction-assisted ureteroscopy are often referred to as the same intervention, masking which design factors and operational characteristics lead to successful performance and safe use of the technology. Therefore, a taxonomy of suction-assisted ureteroscopy should be defined as a ureteroscopic lithotripsy work flow where a suction force is intentionally applied through a sheath-based pathway (i.e., an access-sheath like access conduit), to control the flow of material into the renal collecting system during active fragmentation and/or clearance; suction would then be a controllable factor and not simply an uncontrolled aspiration event (43, 55). There are three additional sub-classifications within this definition to aid in reproducing results. The first sub-classification is based upon distinguishing suction enabled access systems from passive access sheaths used with occasional suction events, since only suction enabled systems provide a continuous collection site capable of altering the intra-renal pressure dynamics and particulate retention time (91). The second sub-classification relates to suction delivery mode, continuous, intermittent or adaptive, since each of these modes create different levels of stability and risk of mucosal contact and varying degrees of field fluctuation (55). The third sub-classification relates to workflow intent, dusting-dominant evacuation, fragmentation-and-evacuation or mixed, since the size distribution of the particles removed and the amount of effluent collected will vary depending on the chosen approach and determine how suction may potentially reduce the residual burden or need for procedural pauses. By standardizing the terminology at these three levels, it will be possible to prevent overgeneralizations, enable comparisons among studies and clarify what is being considered as the “intervention” when clinicians and researchers report outcomes.

### 5.2 Parameter reporting checklist for reproducibility

High translational credibility for high-impact interventions requires that suction-assisted ureteroscopy is described as an intervention that can be reliably replicated rather than as an art based on individual operators. This requires at least a minimal description of four elements of the intervention (access, irrigation, suction, and energy delivery) in each report. The minimum set of information to be reported at baseline includes sheath size and length, location of the intended entry point of the ureteroscope into the ureter (distal ureter, proximal ureter, below the ureteropelvic junction), the type of ureteroscope and its outer diameter, and whether pre-stenting was employed. These factors will determine the feasibility of accessing the desired portion of the ureter and will establish the baseline geometry of outflow from the kidney. To enhance the reproducibility and comparability of research, we suggest that future research reports must go beyond current vague descriptions and clearly report the following parameters: the suction control method employed (vacuum-controlled suction setting or suction controlled by limiting flow), suction method (continual, intermittent, adaptive), and criteria employed to adjust suction (presence of particulate matter in the irrigant fluid, presence of particulate matter in the urine, presence of particulate matter in the calyces, bleeding during lithotripsy). Reporting on irrigation should include inflow method (gravity vs. pump), nominal pump pressure range or gravity height range, and whether the clinical workflow utilizes low inflow rates with suction driven clarity, or higher inflow rates with passive washout. These decisions directly impact how intrarenal pressures are managed and how much thermal energy is buffered during lithotripsy. Reporting on lithotripsy should include type of lithotripsy platform utilized, fiber size, and descriptive information about the energy delivery system sufficient to infer either the intent to produce fragmented stone material or dust. Dust generation rate and heat generated during lithotripsy are directly related to both performance and safety. Reports should also describe the purpose of basket usage, the approach used to remove fragments, and the stenting protocol used after the procedure, as all three are independently associated with the amount of residual fragments and patient-reported symptoms, regardless of suction. Without the minimum reporting set, outcome comparison studies are inherently unreliable due to inability to recreate the intervention, and interpretive analysis is speculative.

### 5.3 Harmonized outcome definitions and measurement standards

There are two major barriers to synthesis based on the heterogeneity of study outcomes: one is based on the clinical outcomes such as stone-free status and infections, and the other is based on the type of treatment (suction vs. laser). For stone-free status, it would be beneficial to establish some explicit residual stone burdens and imaging criteria to replace the implicit notion that there are no symptoms or the surgeon's impression that the patient is stone-free. The imaging modality, time-point and residual stone size threshold need to be stated for each report. To achieve this goal, some studies have constructed predictive models for stone free rates through multivariate analysis, which helps identify key factors that may affect outcomes before surgery and makes the results more clinically significant (92). In addition to stone-free status, ultrasound and plain radiography have low sensitivity for detecting small residual stones, and therefore reports should distinguish between “there were no clinically meaningful residual stones” and “there were no stones visible,” and should state whether CT-based assessments were conducted for any subsets of patients. It would also be useful to report residual stone sizes in strata of size since mechanistic studies suggest that suction is primarily effective at removing very small residual stones and preventing smaller stones that will become clinically active. In addition, recent studies have begun to explore the use of preoperative imaging features, such as renal parenchymal density, to predict postoperative SIRS risk, providing a new tool for standardized identification and outcome analysis of high-risk patients (93). Similarly, the composite score combining stone size and hardness (SMASH score) has also been proven to effectively predict postoperative fever, further demonstrating the necessity of developing standardized preoperative risk assessment tools (94).

Treatment failures should be reported as events occurring within predefined follow-up windows as opposed to as treatment failures occurring over undefined intervals. To avoid misclassifying planned staged procedures as treatment failures, it may be beneficial to require standard definitions for treatment failure. Infectious complications should be reported separately by consensus criteria for post-operative fevers, SIRS and sepsis, and in addition to defining the antibiotics used during the initial hospital admission, the culture strategies (urine cultures pre-operatively, intra-operatively, and if possible, stone cultures) and presence/absence of urinary obstruction, since these factors are the primary determinants of infectious complication rates. Injury to the ureters should be graded according to a recognized grading system. Pain and hematuria should be reported with symptom scales that have been validated in this population and there needs to be explicit documentation of the use of stents, since stent discomfort often dominates the symptom profile of post-operative patients. Standardization of these outcome measures will be essential to allow for valid multi-center comparisons and provide evidence suitable for high-level systematic reviews.

### 5.4 Safety operating envelope and stop-rule logic

Suction-assisted ureteroscopy must be viewed as an option for improving access to the collecting system environment, however this is limited by the “safe zone” in terms of pressure stability, thermal safety and tissue safety. The safe zone in terms of pressure is achieved by maintaining adequate outflow compared to inflow and avoiding the use of high pressure irrigation due to turbidity in addition to the application of excessive suction with inadequate inflow buffering to maintain the field stability and reduce the risk of mucosal injury. The safe zone in terms of temperature is established by providing adequate fluid circulation around the laser-stone interface and ensuring that suction does not mask low fluid circulation conditions through temporarily clearing the field to allow for heat build-up. The safe zone in terms of tissue is achieved by avoiding mucosal contact and reducing the amount of stress placed on the ureter during insertion; repeated suction lock episodes, increasing mucosal contact related bleeding or sustained instability in the field should lead to an immediate reduction in suction and a review of the current approach to flow as opposed to an increase in irrigation pressure. Standardized logic to define stopping points enhances safety and allows for comparison across studies: criteria to abandon sheath placement when resistance indicates high potential for ureteral injury; criteria to stage procedures when clarity in the field cannot be maintained without high irrigation pressures; and criteria to prioritize urinary drainage vs. lithotripsy when purulent efflux or physiological instability indicate unsafe infection physiology. Establishing these stop criteria formally transitions the discourse from the enthusiasm for devices to scientific procedure; this is necessary to establish high impact translational validity.

### 5.5 Core dataset template for registries and multicenter comparability

To create new evidence with greater speed than can be generated through randomization alone, a registry-compatible core dataset should be created which captures those controllable parameters and endpoint data likely impacted by suction. The dataset will need to contain patient risk factors for developing an infection, stone characteristics (i.e., location, burden measurements, obstruction/hydronephrosis), microbiologic environment (i.e., culture data and antibiotic treatment), feasibility of access (i.e., pre-stenting success, sheath placement success and placement depth), suction/irrigation characteristics (i.e., mode, control method, range of operation, and triggers) and lithotripsy characteristics (i.e., platform type and whether intended for dusting or fragmentation). The dataset will require standardized endpoint assessments including the assessment modality and timing for determining if a patient is free from stones, classification of residual fragments, operative time, use of unplanned postoperative care, graded severity of infectious complication(s) and graded severity of ureteral injury(ies). The inclusion of process measures which act as mechanistic intermediate measures — e.g., irrigation volume, visibility recovery pauses — will provide insight into how centers differ in their mechanical execution and will allow for quality improvements prior to establishing superiority in definitive outcomes. A registry compatible core dataset will allow for the development of a comprehensive evaluation of all existing devices and techniques/practices, will aid in the description of learning curve progression and will provide the foundation for future meta-analysis of the effectiveness of the devices and practices in preventing low frequency but high impact events such as sepsis and hospital readmission.

## 6 Discussion, limitations, future directions, and conclusion

When ureteroscopy is viewed through a systems lens, suction-assisted ureteroscopy should be considered a systems-based approach to re-engineering the intra renal environment (42, 95–97). This view supports a mechanistic interpretation of how suction assisted ureteroscopy can provide clinically relevant benefits across diverse practice environments. Specifically, active suction evacuation can reduce the residence time of turbid effluent and suspended particles, thereby allowing for better stabilization of the visual field, and providing for more deliberate, continuous lithotripsy. Additionally, improved outflow will help reduce the need to increase irrigation pressures to regain visibility, resulting in reduced peak pressure excursions that could create pathways for backflow of contaminated fluids (98, 99). Finally, reduction of “visibility recovery” times will result in shorter overall operative times, which will in turn reduce patient exposure to anesthesia, laser heat loads, and procedural manipulation. The integration of these concepts will also explain why suction-assisted ureteroscopy is likely to have non-uniform benefits across all stone scenarios. In the case of low burden ureteral stones where the primary treatment modality has been straightforward fragmentation and extraction, conventional ureteroscopy is likely performing at a near ceiling level of efficacy, and therefore the incremental utility of suction-assisted ureteroscopy will be primarily related to workflow optimization and not to outcomes. In contrast, in the case of intrarenal stones where the primary treatment modality involves significant amounts of dusting (especially when there is a high amount of stone burden, complex calyceal distribution, or expected turbidity), suction-assisted ureteroscopy can directly impact the ability to clear the stone fragments and visualize the operative site, thus making suction's mechanisms directly relevant to the completion of the procedure and the efficiency of the procedure (100–102). Thus, based on this mechanistic and system-based understanding of suction-assisted ureteroscopy, the implications for clinical practice will be conditional and selection sensitive. Suction-assisted ureteroscopy will best be positioned as an optimization strategy for high-dust, high-visibility demand cases, and for physiological environments in which the ability to contain pressure is of highest priority, provided that ureteral access is atraumatic, and that suction – irrigation coupling is safely managed.

The primary limitations on firm inference and on synthesizing the literature with certainty relate to methodology, not concepts. In terms of methodology, first, there is still considerable variation among interventions (devices) related to sheath configuration and sealing mechanisms, variations in how suction is delivered (pressure based vs. flow limited), and differences in intraoperative practices such as irrigation escalation, suction duty cycling, and laser use that have been documented with insufficient detail to allow for mechanistic adjustments. Additionally, second, there is significant variability in the measurement of outcomes that limits the ability to compare results; stone free status is typically measured using differing threshold criteria and is assessed through a variety of imaging methods at varying time points and often is reported without stratifying by residual fragment size that are most important to understanding the impact of suction on fine residual burden. Third, third-party confounding by indication is present in the use of suction in the clinical environment; suction is often used in conjunction with procedures that are considered to be of higher complexity and/or risk. These factors can mask potential benefits associated with suction in unadjusted analyses. Furthermore, the use of suction can produce misleading evidence of benefit when it is employed by more experienced surgeons or in institutions that possess superior perioperative care processes. Fourth, fourth, the measurement of infection-related outcomes is especially difficult due to inconsistencies in the reporting of antibiotic protocols, culture strategies, and the definition of fever, systemic inflammatory response syndrome (SIRS), and sepsis, and because the physiological pathway most likely impacted by suction (backflow under pressure within contaminated systems) is infrequently measured directly; this creates a gap between the proposed mechanism of action and the attribution of outcome. Lastly, fifth, there is a lack of standardized grading of ureteral injuries and the reporting of mucosal apposition phenomena and thermal safety contexts in the reporting of adverse events related to suction, despite the fact that the selection of suction and irrigation modes directly influence the occurrence of these types of adverse events. Therefore, collectively, the above-mentioned limitations suggest that the next step forward in the field of urology will come from increasing standardization in research design that enables results to be compared and synthesized across centers and devices.

Based on the limitations of the methodology and evidence mentioned above, we propose the following priority research agenda to promote the development of evidence to a higher level in this field. The first area of emphasis will be standardizing the reporting of suction and irrigation parameters (including suction delivery mode, control methods, operational range and the specific irrigation method and intensity used) along with descriptors of laser technique sufficient to estimate dust production and heat loads so that the “active agent” of suction assistance can be recreated from study data. The second area of emphasis will be incorporating intrarenal pressure measurements or valid surrogate measures in prospective cohorts to allow for determination of the relationship between flow states, pressure spikes, and infectious outcomes as a way of establishing empiric threshold values for clinically meaningful interventions. The third area of emphasis will be developing uniformity in the measurement of outcomes. Stone-free status assessments should include imaging modality and time-point, as well as a description of residual fragment size. Infection endpoints should use consensus definitions and document perioperative antibiotic and culture usage to allow for reliable meta-analysis. The fourth area of emphasis will be development of pragmatic multicenter registries with a common set of variables describing both the level of detail regarding the intervention (parameter-level), as well as the occurrence of event-based outcomes (e.g., unplanned admission, readmission, retreatment) that are relatively uncommon in single center studies, but represent the most important determinants of value for patients. The fifth area of emphasis will be the development of innovative devices and workflows that utilize computational and experimental fluid dynamics to develop new designs that reduce the risk of mucosal apposition, maintain consistent clearance in dependent calyces, and ensure thermal safety without encouraging low-flow states. Finally, the cost-effectiveness of suction systems will need to be determined using realistic estimates of operating room time and complication costs since the primary rationale for adopting suction systems is likely to be due to the potential to save time and avoid high-cost complications associated with infections rather than minimal improvements in stone-free rates.

## 7 Conclusion

Negative-pressure suction sheath – assisted ureteroscopy, has clinical relevance, in terms of evolving endoscopic stone surgery because it addresses many intraoperative challenges that are most common reasons for limiting the effectiveness and safety of ureteroscopic procedures: visual instability caused by high-dust fragmentation; ineffective particulate removal that leaves behind residual fragments; and, the potential for increased infectious risk due to variable pressure to patients who are susceptible. Suction-assisted ureteroscopy redefines ureteroscopy, as a controlled flow system with irrigation, evacuation, and energy delivery coupled to provide a consistent optical and physiologic micro-environment; as opposed to using intermittent flushing and increasing irrigation pressures in response to changes in the micro-environment. When suction-assisted ureteroscopy is performed within an operating discipline — (atraumatic access); (parameterized suction delivery); (adequate thermal buffering via sufficient fluid turnover); (explicit stop-rule logic) — this modality is better suited to optimize procedural efficiency and reduce variability in effective clearing of particles in intrarenal, high-dusting situations and environments where pressure control is a priority. The present state of knowledge is restricted due to heterogeneity in the architecture of the devices, variability in the reported parameters of suction and irrigation, variability in how “stone free” status and remaining fragments are defined and the varying quantification of the risks of infection and the perioperative use of antibiotics. These issues can be resolved and should be recognized as the next goal for the field: develop a standardization of nomenclature for interventions and a minimum data set to report; develop consistent definitions of outcomes and events; and include physiologic monitors and process metrics in prospective multicenter registry studies and pragmatic clinical trials. By taking these steps, suction-assisted ureteroscopy will move beyond being a new technique with potential to being a reproducible practice that provides evidence based assessment of its effect on the number of remaining fragments, rate of infectious complications, unplanned care use and overall economic utility in managing urinary tract stones today.
